# A cross-sectional study to characterize local HIV-1 dynamics in Washington, DC using next-generation sequencing

**DOI:** 10.1038/s41598-020-58410-y

**Published:** 2020-02-06

**Authors:** Keylie M. Gibson, Kamwing Jair, Amanda D. Castel, Matthew L. Bendall, Brittany Wilbourn, Jeanne A. Jordan, Keith A. Crandall, Marcos Pérez-Losada, the DC Cohort Executive Committee, Thilakavathy Subramanian, Jeffery Binkley, Rob Taylor, Nabil Rayeed, Cheryl Akridge, Stacey Purinton, Jeff Naughton, Natella Rakhmanina, Larry D’Angelo, Michael Kharfen, Angela Wood, Michael Serlin, Princy Kumar, David Parenti, Alan Greenberg, Anne Monroe, Lindsey Powers Happ, Maria Jaurretche, James Peterson, Ronald D Wilcox, Sohail Rana, Michael A Horberg, Ricardo Fernández, Annick Hebou, Carl Dieffenbach, Henry Masur, Jose Bordon, Gebeyehu Teferi, Debra Benator, Maria Elena Ruiz, Deborah Goldstein, David Hardy

**Affiliations:** 1 Computational Biology Institute, The Milken Institute School of Public Health, The George Washington University, Washington, DC 20052 USA; 2 Department of Epidemiology, The Milken Institute School of Public Health, The George Washington University, Washington, DC 20052 USA; 3 Department of Biostatistics and Bioinformatics, The Milken Institute School of Public Health, The George Washington University, Washington, DC 20052 USA; 4 CIBIO-InBIO, Centro de Investigação em Biodiversidade e Recursos Genéticos, Universidade do Porto, Campus Agrário de Vairão, Vairão, Portugal; 5 Cerner Corporation, Arlington, VA 22209 USA; 6 Children’s National Medical Center Pediatric Clinic, Washington, DC 20010 USA; 7 Children’s National Medical Center Adolescent Clinic, Washington, DC 20010 USA; 8 DC Department of Health HAHSTA, Washington, DC 20002 USA; 9 Family and Medical Counseling Service, Washington, DC 20020 USA; 10 Georgetown University, Washington, DC 20007 USA; 11 George Washington Medical Faculty Associates, Washington, DC 20037 USA; 12 Howard University Hospital Adult Clinic, Washington, DC 20059 USA; 13 Howard University Hospital Pediatric Clinic, Washington, DC 20060 USA; 14 Kaiser Permanente, Rockville, MD 20852 USA; 15 La Clinica Del Pueblo, Washington, DC 20009 USA; 16 MetroHealth, Washington, DC 20005 USA; 17 National Institutes of Health, Bethesda, Maryland 20892 USA; 18 Washington Health Institute, Washington, DC 20017 USA; 19 Unity Health Care, Washington, DC 20019 USA; 20 Veterans Affairs Medical Center, Washington, DC 20422 USA; 21 Washington Hospital Center, Washington, DC 20010 USA; 22 Whitman-Walker Health, Washington, DC 20036 USA

**Keywords:** Genome informatics, HIV infections

## Abstract

Washington, DC continues to experience a generalized HIV-1 epidemic. We characterized the local phylodynamics of HIV-1 in DC using next-generation sequencing (NGS) data. Viral samples from 68 participants from 2016 through 2017 were sequenced and paired with epidemiological data. Phylogenetic and network inferences, drug resistant mutations (DRMs), subtypes and HIV-1 diversity estimations were completed. Haplotypes were reconstructed to infer transmission clusters. Phylodynamic inferences based on the HIV-1 polymerase (*pol*) and envelope genes (*env*) were compared. Higher HIV-1 diversity (n.s.) was seen in men who have sex with men, heterosexual, and male participants in DC. 54.0% of the participants contained at least one DRM. The 40–49 year-olds showed the highest prevalence of DRMs (22.9%). Phylogenetic analysis of *pol* and *env* sequences grouped 31.9–33.8% of the participants into clusters. HIV-TRACE grouped 2.9–12.8% of participants when using consensus sequences and 9.0–64.2% when using haplotypes. NGS allowed us to characterize the local phylodynamics of HIV-1 in DC more broadly and accurately, given a better representation of its diversity and dynamics. Reconstructed haplotypes provided novel and deeper phylodynamic insights, which led to networks linking a higher number of participants. Our understanding of the HIV-1 epidemic was expanded with the powerful coupling of HIV-1 NGS data with epidemiological data.

## Introduction

Despite recent reductions in HIV-1 prevalence in Washington, DC from 2.5% in 2013^1^ to 1.8% in 2018, the United States (US) capital is still experiencing a generalized HIV-1 epidemic – as defined by the World Health Organization^2–4^. There were 340 newly diagnosed cases in DC in 2018, and the DC rate is five times higher than the national rate^3^. Blacks, men, men who have sex with men (MSM), and heterosexuals (HRH) account for the majority of people living with HIV-1 (PLWH) in DC^2,3^. However, ~20% of the newly diagnosed persons had an unknown risk for transmission in both 2016 and 2017^2,3^. Furthermore, the leading group (33.3%) of newly diagnosed cases was between the ages of 20–29 years old^3^. This same age group had the highest percentage (27.5%) of drug resistance mutations (DRM) at diagnosis, suggesting broader spread of HIV-1 drug resistant variants and potential concern for future therapeutic options, especially if these mutations are against first line antiretroviral (ART) drugs for newly infected individuals. With blacks and young adults being the most impacted groups of individuals for HIV-1 in DC, understanding the current HIV-1 phylodynamics can provide informative data to guide programs that prevent and reduce the incidence of HIV-1. Moreover, identifying potential transmission clusters amongst individuals in DC and their associated epidemiological features may help infer otherwise ‘unknown’ transmission modes and provide insight for more targeted prevention and intervention strategies.

In 2011, the DC Cohort, a longitudinal observational NIH-funded cohort study of PLWH who are receiving care at clinical sites in DC, began enrollment. As of 2018, the Cohort has enrolled approximately 10,000 PLWH^2^. By capitalizing on the longitudinal study of the DC Cohort, phylodynamics (i.e., the study of how epidemiological, immunological, and evolutionary processes act and potentially interact to shape viral phylogenies^5^) can provide insights into HIV-1 infection in DC. Analyzing sequence data can detect new variants within the population, identify population structuring and associations with risk factors, and, in combination with demographic information, predict areas of interest to direct public health efforts.

The great power and resolution of Next-Generation Sequencing (NGS) technologies are changing phylodynamics research. NGS is used for active infectious disease surveillance^6^, detection of circulating drug resistant variants^7,8^, and inference of HIV-1 transmission clusters. High variation among viral strains of RNA viruses such as HIV-1 are a result of high mutation rates, large population sizes, and short generation times^9^. NGS can detect mutations present in less abundant strains (<1%)^8^. Such rare mutations are particularly relevant in the context of the evolution of drug resistance, since they may facilitate viral adaptation leading to treatment failure^10,11^. Moreover, sequence variants (or haplotypes) can be reconstructed from NGS sequencing reads. Viral populations may contain a pool of different variants that are resistant to different antiretroviral drugs^12,13^ and also help the virus to evade the immune system^14^. Reconstructing the haplotypes present in a viral sample and assessing their phylodynamics may show additional or different transmission clusters present between individuals or identify a few HIV-1 strains that are dominating the HIV-1 viral population^15–17^. The use of powerful NGS technologies to study the HIV-1 epidemic at local levels (e.g., Washington, DC) may generate deeper insights into the ongoing HIV-1 dynamics. Near full length sequences and amplicon sequences that span entire HIV-1 genes are becoming more prevalent with this advanced technology^18,19^. Some studies have indicated that *pol* is less informative than *env* for phylogenetic resolution^20^. As e*nv* evolves at a faster rate than *pol*^19,21^, *env* has shown to be useful in determining the recency of HIV-1 acquisition^22^ and could provide more resolution to infer active or recent transmission clusters than *pol*.

This study applies NGS to a subset of newly and previously diagnosed participants in the DC Cohort to characterize the recent (2016 and 2017) local phylodynamics of HIV-1 in Washington, DC. Towards this general aim, we 1) estimate the diversity of HIV-1 in Washington, DC, 2) determine the circulating drug resistant mutations, 3) identify and evaluate potential transmission clusters with consensus sequences and their association with epidemiologic and clinical factors, and 4) predict HIV-1 haplotypes for each sample and assess their potential for detecting transmission clusters. The number and size of transmission clusters may vary across HIV-1 gene regions^12,15,23–25^, hence in this study we also compared phylodynamic inferences based on the polymerase and envelope HIV-1 genes.

## Results

### Sample and phenotypic characterization

Our sampling included PCR products from 68 participants in the DC Cohort. Most of the study participants resided in Washington, DC (Table 1). The majority were non-Hispanic black (82.4%) and male (69.1%), with 52.9% of participants being non-Hispanic black males. The majority of participants were infected through heterosexual sexual contact (39.7%) followed closely by MSM sexual contact (35.3%). A total of 76.4% of the patients were on an ART drug regimen at the time of blood sample collection. The demographics of our subsample of DC Cohort participants reflects a similar composition of PLWH in DC^3,4^ but includes slightly more participants infected through heterosexual contact and non-Hispanic Blacks, and a lower proportion of participants on ART than the overall Cohort sample.

**Table 1:** Demographic and clinical characteristics for DC Cohort participants whose samples were sequenced and passed filtering criteria.

	Total N = 68
**Age Range**	
20–29 yrs	7
30–49 yrs	31
50–69 yrs	30
**Median Age (yrs, IQR)**	46.3 (23.5, 66.8)
**Race/Ethnicity**	
Non-Hispanic Black	56 (82.4%)
Non-Hispanic White	4 (5.9%)
Hispanic	5 (7.4%)
Unknown	3 (4.4%)
**Sex at Birth**	
Male	47 (69.1%)
Female	21 (30.9%)
**Gender**	
Male	45 (66.2%)
Female	21 (30.8%)
Transgender	1 (1.5%)
Unknown	1 (1.5%)
**Country of Birth**	
US	59 (86.8%)
Non-US	6 (8.8%)
Unknown	3 (4.4%)
**State of Residence**	
DC	55 (80.9%)
MD	10 (14.8%)
VA	3 (4.4%)
**HIV-1 Risk Factor**	
MSM	24 (35.3%)
IDU	6 (8.8%)
HRH	27 (39.7%)
UNK	11 (16.2%)
**Co-infections**^a^	
Syphilis	1 (1.4%)
Hepatitis B	1 (1.4%)
Hepatitis C	1 (1.4%)
**Median Duration of Infection (yrs, IQR)**	12.2 (5, 18)
**Median CD4 count (cells/ul, IQR)**	419.3 (69.5, 586)
**Viral Load Range (copies/ml)**^**b**^	
<200	21 (30.9%)
200–399	5 (7.4%)
400–9,999	14 (20.6%)
>10,000	3 (4.4%)
Unknown	25 (36.8%)
**ART Exposure**	
Experienced	61 (89.7%)
Naïve	7 (10.3%)
**ART Regimen Type**	
Multiple-Class	50 (73.5%)
Dual-Class	2 (2.9%)
Unknown	16 (23.5%)
**Amplicon Presence**	
*Before Quality Filtering*	
*PR/RT*	68 (100%)
*int*	68 (100%)
*env*	62 (91.2%)
*After Quality Filtering*	
*PR/RT*	62 (91.2%)
*int*	62 (91.2%)
*env*	47 (69.1%)

^a^Co-infections were determined to be present within 30 days of sample collection.

^b^Viral load and CD4 count were determined for participant within 30 days of sample collection.

MSM = men who have sex with men; HRH = heterosexuals; IDU = injection drug users; UNK = unknown.

### HIV-1 diversity in DC

We performed in-depth phylodynamic profiling of HIV sequences from 171 PCR gene products that passed quality thresholds, including 62 *PR/RT*, 62 *int*, and 47 *env* amplicons. The subtyping analyses showed that all of the participants belonged to subtype B; therefore, subsequent analyses included data from all participants. Our participants were dispersed amongst and showed a star-like pattern with other DC HIV *PR/RT* sequences (see Supplementary Fig. S1 online). The *env(c)* data showed higher nucleotide diversity (π) and Watterson genetic diversity (θ) than *pol(c)* (Table 2). Males had a higher diversity, though not significant, than females (haplotype diversity: *PR/RT*: p = 0.2039, *int*: p = 0.9571, *env*: p = 0.3404). Participants whose risk factor was IDU (n = 6) had 50% less diversity than those with MSM and HRH risk, though again not significant (haplotype diversity: *PR/RT*: p = 0.7323, *int*: p = 0.7861, *env*: p = 0.6560). Non-Hispanic black participants had a higher genetic diversity for the *pol(c)* gene than for the *env(c)* gene (Table 2). The average haplotype diversity when calculated with the number of reconstructed haplotypes by PredictHaplo showed that *env* had more haplotype diversity compared to *PRRT* and *int* (Table 3). The average number of haplotypes per participant was the same for *PR/RT* and *int* (2 haplotypes) and slightly higher for *env* (4). Four of the six participants that had a higher number of haplotypes reconstructed (7–12 haplotypes in one or more gene regions) also had a higher average haplotype diversity estimate of 0.634 (range: 0.392–0.777), while the other two had a very low average haplotype diversity estimate (0.032, range: 0.027–0.038). Participants with HRH and MSM risk factors were found to have an average of 3 reconstructed haplotypes, with haplotype diversities of 0.338 and 0.335, respectively.

**Table 2:** Nucleotide diversity between the *pol* and *env* concatenated consensus sequences.

	Diversity					DRM
	N	S	h	π	θ (W)	
***pol***	68	702	68	0.051	0.079	40.0%
***Risk Factors***						
MSM	24	450	25	0.051	0.070	33.3%
HRH	25	321	26	0.051	0.070	48.0%
IDU	6	138	6	0.034	0.034	66.7%
***Sex***						
Male	47	583	49	0.049	0.075	53.2%
Female	20	408	20	0.051	0.066	45.0%
***Race/ethnicity***						
Non-Hispanic Black	55	608	56	0.050	0.076	47.3%
Non-Hispanic White	4	150	4	0.060	0.060	50.0%
Hispanic	5	180	6	0.046	0.048	60.0%
***env***	47	489	47	0.228	0.202	
***Risk Factors***						
MSM	18	402	19	0.227	0.213	
HRH	14	371	15	0.226	0.214	
IDU	6	196	6	0.164	0.162	
***Sex***						
Male	35	475	37	0.234	0.218	
Female	12	328	12	0.219	0.202	
***Race/ethnicity***						
Non-Hispanic Black	38	463	39	0.223	0.206	
Non-Hispanic White	4	210	4	0.219	0.211	
Hispanic	3	203	4	0.217	0.211	

Diversity (N = number of sequences, S = number of segregating sites, h = number of haplotypes, π = nucleotide diversity, θ = Watterson genetic diversity) rates. Total and relative (total/N) proportion (%) of HIV-1 strains including DRM. MSM = men who have sex with men; HRH = heterosexuals; IDU = intravenous drug users.

**Table 3:** Haplotype diversity estimates from PredictHaplo results.

Participant	*PR*/*RT*		*int*		*env*		Viral Load (copies/mL)	ARV Exposure	ARV Regimen Type
	Number of Haplotypes	Haplotype Diversity	Number of Haplotypes	Haplotype Diversity	Number of Haplotypes	Haplotype Diversity			
**8**	NA	NA	1	0	NA	NA		E	2 NRTI + 1 ENH + 1 INSTI
**9**	NA	NA	5	0.066	11	0.038	476	E	
**12**	1	0	4	0.727	2	0.393	77	E	2 NRTI + 1 NNRTI
**13**	NA	NA	1	0	2	0.302		N	
**16**	1	0	4	0.727	2	0.393		E	
**18**	NA	NA	1	0	NA	NA	119,149	E	
**19**	2	0.436	2	0.499	4	0.721	244	N	
**20**	2	0.479	1	0	2	0.281	5,663	E	1 NRTI + 1 PI + 1 ENH
**23**	3	0.352	2	0.484	NA	NA	927	E	2 NRTI + 1 ENH + 1 INSTI
**25**	1	0	1	0	NA	NA		E	2 NRTI + 1 ENH + 1 INSTI
**26**	2	0.452	2	0.466	NA	NA		N	
**27**	1	0	1	0	NA	NA	11	E	1 PI + 1 ENH
**29**	2	0.464	2	0.250	5	0.620	1	E	2 NRTI + 1 ENH + 1 INSTI
**30**	2	0.441	3	0.579	1	0	625	E	
**31**	3	0.64	3	0.526	2	0.312		E	2 NRTI + 1 INSTI
**32**	2	0.146	2	0.339	2	0.498		E	2 NRTI + 1 NNRTI + 1 PI + 1 ENH
**33**	1	0	3	0.563	3	0.601		E	2 NRTI + 1 ENH + 1 INSTI
**34**	2	0.429	2	0.2	1	0		E	2 NRTI + 1 INSTI
**35**	2	0.385	1	0	3	0.446		E	2 NRTI + 1 INSTI
**37**	2	0.274	1	0	10	0.027	81	E	
**39**	NA	NA	1	0	NA	NA		E	2 NRTI + 1 ENH + 1 INSTI
**40**	2	0.494	4	0.692	3	0.623		E	
**42**	1	0	2	0.215	2	0.445	13,979	E	2 NRTI + 1 ENH + 1 INSTI
**43**	1	0	7	0.833	NA	NA	343	E	
**45**	1	0	10	0.704	NA	NA		E	2 NRTI + 1 ENH + 1 INSTI
**46**	2	0.153	3	0.306	2	0.5		N	
**47**	2	0.263	2	0.420	1	0	2,755	E	1 NNRTI + 1 PI + 1 ENH + 1 INSTI
**49**	2	0.455	1	0	2	0.324	282	E	2 NRTI + 1 ENH + 1 INSTI
**50**	1	0	2	0.344	3	0.624	576	E	2 NRTI + 1 PI + 1 ENH
**51**	1	0	1	0	3	0.593		E	2 NRTI + 1 PI + 1 ENH + 1 INSTI
**52**	2	0.439	1	0	NA	NA		E	2 NRTI + 1 PI + 1 ENH
**54**	3	0.583	1	0	2	0.491	412	E	2 NRTI + 1 PI + 1 ENH
**55**	1	0	1	0	2	0.384		E	1 PI + 1 ENH
**56**	12	0.392	3	0.641	NA	NA	1,368	E	2 NRTI + 1 ENH + 1 INSTI
**57**	1	0	1	0	NA	NA	12,786	E	2 NRTI + 1 ENH + 1 INSTI
**58**	1	0	3	0.590	4	0.659	1,281	E	2 NRTI + 1 ENH + 1 INSTI
**59**	3	0.572	1	0	4	0.705	581	E	2 NRTI + 1 INSTI
**60**	2	0.487	3	0.619	4	0.716		E	2 NRTI + 1 PI + 1 ENH
**61**	2	0.270	3	0.632	4	0.679	432	E	
**63**	3	0.612	6	0.708	1	0	57	E	2 NRTI + 1 PI
**64**	5	0.759	3	0.576	4	0.697		E	
**65**	2	0.492	2	0.441	3	0.186		E	2 NRTI + 1 PI + 1 ENH
**66**	1	0	2	0.490	5	0.602	32	E	2 NRTI + 1 ENH + 1 INSTI
**67**	NA	NA	2	0.450	5	0.767		E	2 NRTI + 1 NNRTI
**68**	3	0.564	5	0.736	5	0.758		E	2 NRTI + 1 NNRTI
**69**	1	0	4	0.543	7	0.777	48	E	2 NRTI + 1 PI + 1 ENH
**70**	4	0.603	2	0.364	5	0.743	17	N	
**71**	1	0	4	0.720	6	0.812		E	1 NRTI + 1 NNRTI + 1 INSTI
**72**	2	0.209	3	0.614	NA	NA	353	E	2 NRTI + 1 NNRTI
**73**	1	0	3	0.660	2	0.414		E	2 NRTI + 1 ENH + 1 INSTI
**74**	1	0	5	0.779	4	0.037	1	E	2 NRTI + 1 ENH + 1 INSTI
**75**	1	0	3	0.533	7	0.663		E	2 NRTI + 1 PI + 1 ENH
**76**	1	0	1	0	5	0.777	432	E	2 NRTI + 1 INSTI
**77**	4	0.613	4	0.658	6	0.768	113	E	2 NRTI + 1 PI + 1 ENH + 1 INSTI
**78**	2	0.183	3	0.608	5	0.664	113	E	2 NRTI + 1 PI + 1 ENH
**79**	1	0	1	0	2	0.396		E	2 NRTI + 1 INSTI
**82**	1	0	NA	NA	NA	NA		N	
**83**	2	0.467	NA	NA	NA	NA	554	E	2 NRTI + 1 NNRTI
**85**	2	0.351	1	0	2	0.477	183	E	2 NRTI + 1 INSTI
**86**	2	0.433	1	0	NA	NA		E	2 NRTI + 1 PI + 1 ENH + 1 INSTI
**87**	2	0.499	NA	NA	NA	NA		E	2 NRTI + 1 ENH + 1 INSTI
**88**	1	0	NA	NA	1	0	37	N	
**90**	3	0.534	2	0.498	3	0.540	8,914	E	2 NRTI + 1 PI + 1 ENH + 1 CCR5 + 1 INSTI
**91**	1	0	2	0.003	NA	NA	117	E	2 NRTI + 1 INSTI
**93**	2	0.446	1	0	NA	NA	94	E	2 NRTI + 1 ENH + 1 INSTI
**94**	1	0	NA	NA	NA	NA	145	E	2 NRTI + 1 PI + 1 ENH
**97**	5	0.718	2	0.455	5	0.782		E	2 NRTI + 1 INSTI
**99**	1	0	NA	NA	1	0	184	E	2 NRTI + 1 ENH + 1 INSTI
**Avg**	2	0.265	2	0.357	4	0.470			

A haplotype diversity of 0 indicates no diversity because only a single haplotype was reconstructed by PredictHaplo for the sample. Amplicons that did not pass the filtering thresholds for a sample are indicated by “NA”. ARV exposure is reported at time that blood sample was taken. N: Naïve, E: Experienced, NRTI: Nucleoside reverse transcriptase inhibitors, NNRTI: Non-nucleoside reverse transcriptase inhibitors, ENH: enhancer elements, PI: Protease Inhibitor, INSTI: Integrase Strand Transfer Inhibitor, CCR5: Cysteine-Cysteine Chemokine Receptor 5.

### Drug resistant mutations

The consensus concatenated gene regions (*pol(c)* and *env(c)*; see Methods for definition of “(c)”, which in short stands for consensus) for each participant were used to evaluate the presence of Drug Resistant Mutations (DRMs) (Table 4). The *PR* gene from participants had the fewest DRMs (1), compared to *RT* (25) and *int* (11) genes. The majority of DRMs were NRTI, NNRTI, and RT surveillance DRMS (SDRMs). Together, they included 12 to 24 resistant participants and 15 to 44 total DRMs (9 to 19 unique DRMs). Finally, 34 participants (50.0%) showed at least one mutation and 24 (35.3%) showed at least two different DRMs. Of the ARV treatment naïve participants, none were found to have a DRM present in the *PR* and *RT* genes, and only a single participant was found to have an IN Accessory DRM at amino acid 157. This treatment naïve individual with a DRM was a part of a transmission cluster for all genes except the haplotype *V1V2* gene region. An overall DRM prevalence of 1.4%, 35.7%, and 15.7% was estimated for *PR*, *RT*, and *int*, respectively. The 40–49 year-olds in our study had the highest prevalence of DRMs (22.9%), while the 20–29 year-olds in our study did not show any DRMs. Overall, DRMs caused amino acid changes in only one codon position in *PR*, while 22 and 9 different codons positions changed in *RT* and *int*, respectively, when analyzing the consensus sequences. However, more codons were affected by an amino acid change in *PR* (5 codons) and *RT* (27 codons) when analyzing the haplotype sequences. On average, more DRMs and more unique DRMs were identified in the haplotype sequences (Table 4). Also, one young adult (20–29 years old) contained DRMs in *RT*, and one treatment naïve participant contained DRMs in *RT*. Slightly more participants had a haplotype sequence that showed at least one DRM (37 participants; 54.0%) and at least two different DRMs (30; 44.1%).

**Table 4:** Drug Resistant Mutations.

Gene	IN Major	IN Access.	PR Major	PR Access.	NRTI	NNRTI	PR SDRMs	RT SDRMs	PI TSMs	NRTI TSMs	NNRTI TSMs	DRM Codons	FUBAR Codons
**Consensus**													
*PR*	—	—	0/0/0	1/1/1	—	—	0/0/0	—	0/0/0	—	—	1	2
*RT*	—	—	—	—	19/37/18	12/15/9	—	24/44/19	—	1/1/1	1/1/1	22	3
*int*	3/6/6	9/9/3	—	—	—	—	—	—	—	—	—	9	4
*V1V2*	—	—	—	—	—	—	—	—	—	—	—	—	4
*V3*	—	—	—	—	—	—	—	—	—	—	—	—	4
**Haplotypes**													
*PR*	—	—	2/5/3	2/3/2	—	—	2/5/3	—	0/0/0	—	—	5	4
*RT*	—	—	—	—	27/100/19	15/39/13	—	30/117/22	—	3/3/2	1/3/1	27	4
*int*	3/6/5	7/13/1	—	—	—	—	—	—	—	—	—	6	9
*V1V2*	—	—	—	—	—	—	—	—	—	—	—	—	7
*V3*	—	—	—	—	—	—	—	—	—	—	—	—	8

Number of participants/total mutations/unique mutations conferring resistance to antiretroviral drugs (IN Major to NNRTI TSMs) for genes *int* and *PR/RT*. DRM amino acid codons and codons under adaptive selection (FUBAR) are also listed. NRTI: nucleoside reverse-transcriptase inhibitors, NNRTI: non-nucleoside reverse-transcriptase inhibitors, SDRMs: surveillance drug resistant mutations, TSMs: treatment-selected mutations.

FUBAR analysis, which identifies nucleotide positions under positive selection, identified inferred two, three, and four codons under positive selection in the *PR* gene, *RT* gene, and *int* gene, respectively, when analyzing the consensus sequences (Table 4). Positively selected sites 37 and 57 were inferred by FUBAR for *PR* and sites 35, 83, and 162 for *RT*. Amino acid positions 201, 216, 265, and 283 were found to be under positive selection for *int*. No codons overlapped between the FUBAR analysis and DRM analysis for any gene. None of the sites predicted by FUBAR are known resistance sites^26^, suggesting DRMs are fixed in the population. Additionally, FUBAR also found four codons under positive selection for the *V1V2* and *V3* genes (Table 4). These codon sites were 8, 15, 34, 53 for *V1V2* and 82, 90, 93, 106 for *V3*. Every IDU participant had a mutation in at least one of the sites predicted by FUBAR in the *V1V2* and *V3* genes. HRH (55.6%) and MSM (72.0%) participants had a high prevalence of sites under selection as well. Additionally, a total of 51.4% and 17.1% of the male and female participants, respectively, contained a mutation at one of these predicted sites. Furthermore, FUBAR analysis inferred four codons under positive selection in the *PR* and *RT* genes and nine codons for *int* from the haplotype sequences. These sites were 64, 72, 77, and 93 for *PR*; 35, 85, 102, and 200 for *RT*; and 201, 206, 211, 218, 230, 256, 265, 283, and 285 for *int*. The only codon position predicted by FUBAR in the haplotypes was 230 for *int*, and it is associated with reduced susceptibility to integrase inhibitors (INSTI)^27^. Seven (4, 34, 41, 43, 44, 45, 64) and eight (22, 40, 84, 92, 93, 95, 105, 111) codons were inferred to be under positive selection for *V1V2* and *V3* haplotype sequences, respectively. Interestingly, only a handful of inferred positively selected codons overlapped between the haplotype and consensus sequences, those were at position 35 for *RT*; positions 201, 265, and 283 for *int*; 34 for *V1V2*; and 93 for *V3*.

### Transmission clusters

**Figure 1:**
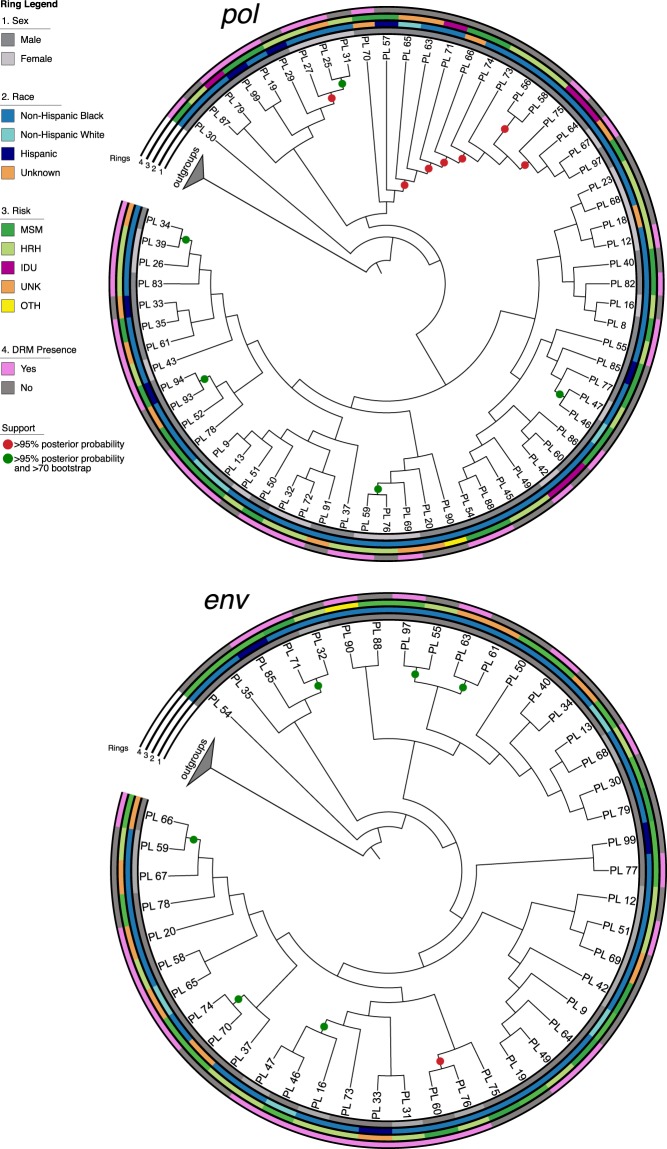
Cladogram of the *pol* and *env* concatenated genes of Washington, DC showing sex, race/ethnicities, and risk factors in rings. All phenotypes present are represented with different colors, see legend. All sequences were subtype B. Well-supported clades are depicted. MSM = men who have sex with men; HRH = heterosexuals; IDU = injection drug users; UNK = unknown; OTH = other. Numbers correspond to the de-identified participant.

Transmission clusters were assessed by phylogenetic methods and HIV-TRACE, a genetic-distance based clustering method. Phylogenetic methods found support (>70% bootstrap or >0.95 posterior probability) for 33.8% of the sequences associated with six transmission clusters in *pol(c)* and for 31.9% of the sequences associated with seven clusters in *env(c)* (highlighted in Fig. 1). All of the clusters were comprised of two to three sequences, except one cluster in *pol(c)* which had twelve sequences. Ten of the twelve sequences in the large *pol(c)* cluster contained a DRM within *RT*. Furthermore, 11 of the 12 sequences in this large cluster were included on the same sequencing run; therefore, we were unable to undoubtfully discriminate between laboratory artifacts or batch effects and HIV infection to interpret this transmission cluster. However, there were other samples from that same sequencing run that did not cluster with these twelve sequences and formed a dyad cluster. The most common DRM was T215C, which does not reduce NRTI susceptibility, and was found in eight of the twelve sequences.

**Figure 2:**
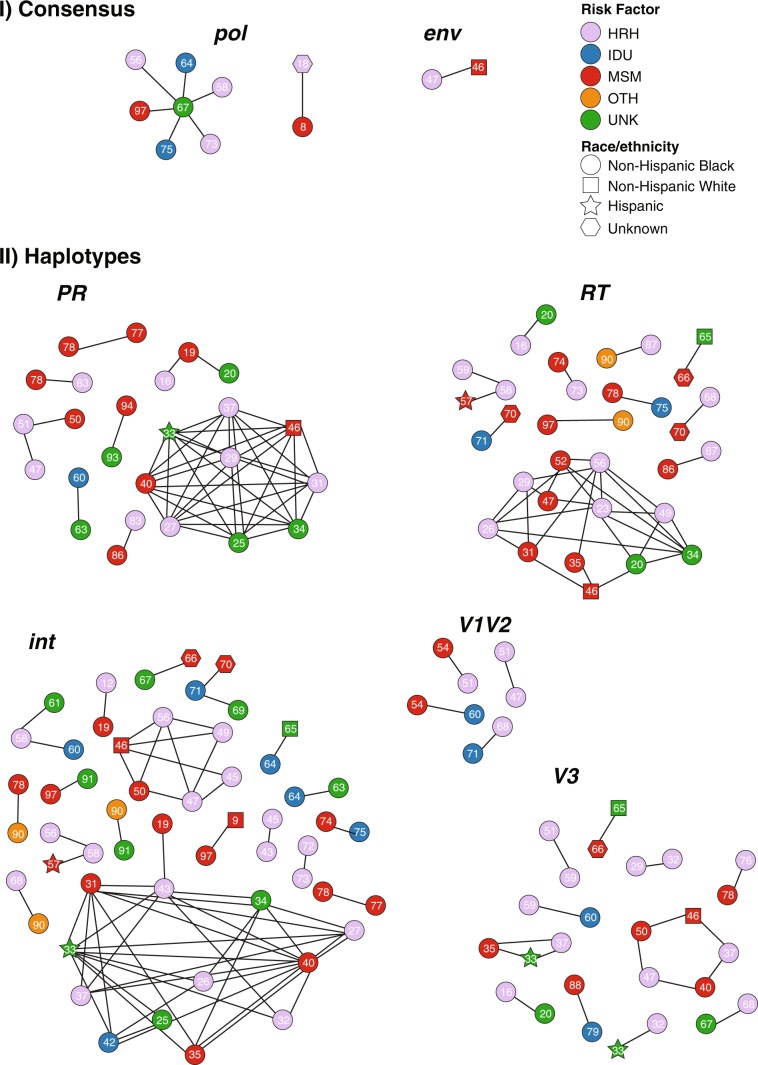
Cluster network (HIV-TRACE) of (I) consensus *pol* and *env* genes and (II) haplotypes reconstructed with PredictHaplo for *PR, RT, int, V1V2* and *V3* genes of Washington, DC participants by risk factor and race/ethnicity. Numbers correspond to the de-identified participant. Some participants have multiple HIV-1 haplotypes.

HIV-TRACE grouped only a few sequences into two clusters for *pol(c)* (12.8%) and into a single cluster for *env(c)* (2.9%) (Fig. 2I). More transmission clusters were estimated with the haplotypes reconstructed from PredictHaplo (Fig. 2II, Table 5). For *PR, RT*, and *int* (genes also used in past DC HIV studies^28,29^), 82.1% of our participants were incorporated into transmission clusters. Unique to our dataset was the use of envelope to predict transmission clusters; 35.8% of our participants were included in *V1V2* and *V3* transmission clusters. Haplotypes were not concatenated for this analysis, but little overlap of cluster composition was found between gene regions. Often one participant’s haplotype would cluster with another participant in one gene region, but it would cluster with a different participant in a different gene region. Thus different gene regions displayed different transmission clusters that would not have been detected if one only analyzes a single gene. Clusters predicted in all gene regions were mainly composed of two participants (42 of the 55 total clusters). Likewise, the majority of the transmission clusters predicted by past DC HIV studies^28,29^ were comprised of two or three participants, regardless of the gene region, clustering estimation method, or haplotype/consensus construction approach.

**Table 5:** Characteristics of transmission clusters with HIV-TRACE and comparison between different genes.

Gene region	Seq type^a^	Cluster Name^b^	N^c^	Sex		Risk factor^d^				Avg Age^e^	DRMs^f^	Overlap between genes, same seq type^g^
				Male	Female	MSM	HRH	IDU	UNK			
*pol*	Cons	Tra_POL_a_7	7	6	1	1	3	2	1	42.3	71%	0%
*pol*	Cons	Tra_POL_b_2	2	1	1	1	1	0	0	37.1	50%	0%
*env*	Cons	Tra_ENV_a_2	2	1	1	1	1	0	0	48.6	100%	0%
*PR*	Haps	Tra_PR_a_9	9	6	3	2	4	0	3	42.5	44%	100%
*PR*	Haps	Tra_PR_b_3	3	2	1	1	1	0	1	58.0	67%	100%
*PR*	Haps	Tra_PR_d_3	3	1	2	1	2	0	0	44.0	67%	100%
*PR*	Haps	Tra_PR_e_2	2	2	0	2	0	0	0	34.5	50%	100%
*PR*	Haps	Tra_PR_f_2	2	2	0	1	1	0	0	38.8	0%	50%
*PR*	Haps	Tra_PR_g_2	2	2	0	1	0	0	1	40.5	50%	0%
*PR*	Haps	Tra_PR_h_2	2	2	0	0	0	1	1	49.9	100%	100%
*PR*	Haps	Tra_PR_i_2	2	2	0	1	1	0	0	39.6	0%	50%
*RT*	Haps	Tra_RT_a_12	12	8	4	3	7	0	2	43.4	67%	83%
*RT*	Haps	Tra_RT_b_3	3	2	1	1	2	0	0	44.2	67%	100%
*RT*	Haps	Tra_RT_c_2	2	2	0	1	0	1	0	44.7	50%	100%
*RT*	Haps	Tra_RT_d_2	2	2	0	1	1	0	0	50.7	100%	100%
*RT*	Haps	Tra_RT_e_2^h^	2	2	0	1	0	0	0	47.8	100%	100%
*RT*	Haps	Tra_RT_f_2^h^	2	2	0	0	1	0	0	59.2	100%	50%
*RT*	Haps	Tra_RT_g_2	2	2	0	1	0	1	0	45.8	0%	100%
*RT*	Haps	Tra_RT_h_2	2	2	0	1	0	0	1	46.5	100%	100%
*RT*	Haps	Tra_RT_i_2	2	1	1	1	1	0	0	47.7	50%	100%
*RT*	Haps	Tra_RT_j_2	2	1	1	0	1	0	1	55.4	100%	100%
*RT*	Haps	Tra_RT_k_2	2	0	2	0	1	0	1	63.7	50%	100%
*int*	Haps	Tra_INT_a_13	13	6	7	3	6	1	3	44.4	23%	85%
*int*	Haps	Tra_INT_b_6	6	5	1	2	4	0	0	42.5	83%	83%
*int*	Haps	Tra_INT_c_3	3	3	0	0	1	1	1	44.5	67%	67%
*int*	Haps	Tra_INT_d_3	3	2	1	1	0	1	1	43.0	33%	67%
*int*	Haps	Tra_INT_e_3	3	3	0	1	2	0	0	43.2	100%	100%
*int*	Haps	Tra_INT_f_2^h^	2	1	1	0	1	0	0	59.4	100%	100%
*int*	Haps	Tra_INT_g_2	2	2	0	1	0	0	1	51.0	100%	50%
*int*	Haps	Tra_INT_h_2	2	1	1	1	1	0	0	57.8	0%	50%
*int*	Haps	Tra_INT_i_2	2	2	0	1	1	0	0	40.2	100%	100%
*int*	Haps	Tra_INT_j_2^h^	2	2	0	1	0	0	0	43.2	50%	100%
*int*	Haps	Tra_INT_k_2	2	2	0	2	0	0	0	51.3	50%	50%
*int*	Haps	Tra_INT_l_2	2	2	0	0	0	1	1	55.9	100%	50%
*int*	Haps	Tra_INT_m_2	2	2	0	0	0	1	1	54.7	100%	50%
*int*	Haps	Tra_INT_n_2^h^	2	2	0	0	0	0	1	55.1	100%	50%
*int*	Haps	Tra_INT_o_2	2	1	1	0	2	0	0	39.7	50%	0%
*int*	Haps	Tra_INT_p_2	2	2	0	1	0	1	0	53.4	50%	100%
*int*	Haps	Tra_INT_q_2	2	1	1	0	2	0	0	52.6	50%	50%
*int*	Haps	Tra_INT_r_2	2	2	0	2	0	0	0	34.5	50%	100%
*V1V2*	Haps	Tra_V1V2_a_2	2	1	1	1	1	0	0	52.5	50%	100%
*V1V2*	Haps	Tra_V1V2_b_2	2	2	0	1	0	1	0	48.8	50%	100%
*V1V2*	Haps	Tra_V1V2_c_2	2	0	2	0	2	0	0	51.7	100%	100%
*V1V2*	Haps	Tra_V1V2_c_2	2	1	1	0	1	1	0	63.8	100%	100%
*V3*	Haps	Tra_V3_a_5	5	4	1	3	2	0	0	42.1	60%	100%
*V3*	Haps	Tra_V3_b_3	3	3	0	1	1	0	1	40.2	33%	100%
*V3*	Haps	Tra_V3_c_2	2	1	1	0	2	0	0	45.7	0%	100%
*V3*	Haps	Tra_V3_d_2	2	2	0	1	0	0	1	46.5	100%	100%
*V3*	Haps	Tra_V3_e_2	2	1	1	1	1	0	0	38.7	50%	100%
*V3*	Haps	Tra_V3_f_2	2	0	2	0	1	0	1	63.7	50%	100%
*V3*	Haps	Tra_V3_g_2	2	1	1	0	1	0	1	43.8	50%	100%
*V3*	Haps	Tra_V3_h_2	2	0	2	0	2	0	0	46.1	50%	100%
*V3*	Haps	Tra_V3_i_2	2	1	1	0	1	1	0	42.5	50%	100%
*V3*	Haps	Tra_V3_j_2	2	1	1	0	1	0	1	55.4	100%	100%
*V3*	Haps	Tra_V3_k_2	2	1	1	1	0	1	0	40.9	0%	0%

^a^Cons = consensus; Haps = haplotypes.

^b^Cluster Name: The first part corresponds to method (HIV-TRACE), the second part corresponds to gene, the third part is an arbitrary letter to distinguish individual clusters, and the fourth part corresponds to the number of sequences belonging to the cluster (N).

^c^Number of unique participants within a cluster.

^d^MSM = men who have sex with men; HRH = heterosexuals; IDU = injection drug users; UNK = unknown.

^e^Average age in years.

^f^Percentage of participants within a cluster that contained one or more DRMs.

^g^Overlap was only assessed between the concatenated consensus *pol* and *env* genes and between genes (*PR, RT, int, V1V2*, and *V3*) with transmission clusters generated with haplotypes. Reported as the percentage of unique participants within the cluster that are found in another cluster in a different gene within the same sequence type (consensus vs haplotype sequences). Overlap was not assessed between sequence types.

^h^One participant had a risk factor of other.

## Discussion

Collectively, our study aimed to characterize the local and recent phylodynamics of a subset of DC Cohort participants from Washington, DC metro area living with HIV. By combining clinical and behavioral data with NGS data, we were able to identify transmission clusters across groups with different demographics and risk behaviors. Additionally, this study reconstructed sequence variants present within a participant (i.e., intra-host) and investigated associations between the reported participant characteristics and transmission clusters.

Our population genetic estimators indicate that HIV-1 *env(c)* is more genetically diverse than *pol(c)*. Similar diversity estimates were found in other studies^19,29–31^. Our estimate of genetic diversity for *pol(c)* in DC (θ = 0.079) was lower than those reported for subtype B in Pérez-Losada *et al*.^29^ (θ = 0.084 and 0.090), but higher than those currently reported for the US subtype B sequences in Los Alamos HIV-1 database (θ = 0.075 for *int*; θ = 0.067 for *PR/RT*). This same trend was seen in *env(c)*, where our DC HIV-1 genetic diversity (θ = 0.202) was greater than that reported for *V1*-*V3* from Los Alamos HIV-1 database (θ = 0.168) across the US. Haplotype diversity estimated from the PredictHaplo results also found *env* genes more genetically diverse than *PR/RT* and *int*.

Notably, there were interesting differences in diversity estimates among risk groups. Participants who were infected through injection drug use (IDU) were found to have about half of the diversity of MSM and HRH participants. This low diversity, potentially associated with low multiplicity of HIV-1 infection, is not unusual within IDU individuals^32,33^. In both *pol(c)* and *env(c)*, males also showed higher genetic diversity, which could be attributed to half (51%) of the males in this study being infected through sex with other men (16% of the males had an unknown risk factor). Our measurements of HIV-1 diversity in HRH were also high and similar to those of MSM; however, we did not observe differences in HIV genetic diversity by race or ethnicity.

The HIV-1 subtype B epidemic in DC is highly diverse, and our results here agree with previous conclusions suggesting a mature epidemic^29,34^. High genetic diversity could result from risk groups intermingling and viral strains being exchanged and the transient nature of the DC metro area population. DC is an international stop for some, a temporary residence for others, and home for many. This constant influx of incomers could have a boosting effect on the DC HIV-1 population by consistently introducing new viral strains into the pool. Treatment and vaccine development can be compromised by high genetic diversity. As HIV-1 continues to evolve and, as seen here, high genetic diversity levels are kept constant over time^34^, resistance to vaccines and ART drugs may increase, which could ultimately lead to treatment and prevention failures^35^.

A Drug Resistant Mutation (DRM) prevalence of 48.6–54.0% was detected in this subset of the DC Cohort, depending on use of consensus sequence or haplotypes. Lower DRM prevalence rates were previously reported for the DC area^28,29,34,36^ (17.3–37.9% between 1994 and 2016). A much higher rate (66%) was reported in a smaller study of ART treatment-naïve and experienced pediatric patients in Rhode Island^37^. DRM rates over 50% are also seen in large sequence databases in the UK and Switzerland^29^. Our study found fewer codons affected by a DRM and fewer DRMs in our participants than previous studies^28,29^ of the DC epidemic. We only found 32 and 38 codons to be affected when analyzing consensus sequences and haplotypes, respectively, whereas Pérez-Losada *et al*.^29^ found 83 codons affected for subtype B, however that study contained 20 times more sequences than ours. Moreover, our study found three novel DRM sites that were not identified in either previous study: P145PAST (IN Major) and A128APST, Q146QH (IN Accessory).

More recently, a study by Kuhnert and colleagues^38^ reported on the fitness of fourteen HIV-1 resistance mutations, of which seven were detected in *RT* in our study. Three of the seven DRMs (codons: 41 L, 67 N, 184 V) were NRTI-related and the other four DRMs (codons: 103 N, 108I, 138 A, 181 C) were NNRTI-related. Six DC Cohort participants contained the 184 V DRM, which was found by Kuhnert *et al*.^38^ to have the highest transmission cost – i.e., the success (low transmission cost) or lack thereof (high transmission cost) of transmission of hosts infected by drug resistant strains. Because of this high cost to the virus, the mutation resulted in very short transmission chains despite evolving frequently under treatment failure^38^. Of the six DC Cohort participants, all were included in a cluster when using the haplotypes, but none of the participants clustered with each other. Additionally, this mutation was found to be persistent in the DC HIV-1 viral population since 2005^34^. Previous studies showed that the most important NNRTI mutation currently is 103 N because of its connection to first-line treatment failure^39–41^; a quarter of our DC Cohort participants with one or more DRMs contained this mutation, which was also found to be at a low frequency in the DC HIV-1 viral population since 2005^34^.

In treatment-naïve participants, both when using consensus sequences and haplotypes, we estimated a low prevalence rate of DRMs (14.3%). Similarly, low prevalence rates have been seen in the past in the US (^42^; 15% between 1999 and 2011) and even lower in treatment-naïve individuals in Europe (^41,43–45^; 10% between 2001 and 2013). Moreover, we also found fewer DRMs in treatment-naïve participants compared to a recent study of PLWH in DC^28^ (22.5% between 1994 and 2013). Kassaye *et al*.^28^ also observed a downward trend of overall prevalence of DRMs over time in treatment-naïve individuals. Our results suggest a further decrease in overall prevalence of DRMs in the current DC HIV-1 epidemic. A lower prevalence of DRMs in surveillance versus targeted treatment-naïve studies could result from sampling design. A surveillance study of DC would likely provide the more accurate picture of DRM trends in the population.

Novel sites that continue to evolve in the DC epidemic and have not become fixed in the population are of serious concern for future drug therapy and conferring resistance to these drugs. More and different codons were predicted to be under positive selection in the haplotype sequences than the consensus sequences. FUBAR predicted sites in *V1V2* are likely being impacted by the immune system, which the virus is actively trying to evade (diversifying selection). *V3* is associated with co-receptor binding^46^; codons predicted here could be rising advantageous mutations by HIV-1 to adapt to the host cells’ response against the virus. Five codons (*PR:* 37, *RT*: 35, and *int*: 201, 265, 283) were identified by both our study, in both haplotypes and consensus sequences, and Pérez-Losada *et al*.^29^ as sites under selection for subtype B. Since none of these sites corresponded to any known Stanford DRMs, these may be newly evolving resistance mutations in the DC HIV-1 epidemic. Given that all of our participants were on dual- or multiple-drug regimens, these sites may also be indicative of potential escape mechanisms by the virus in response to multiple-drugs treatments. Thus, these amino acid replacements are candidates for fitness testing with and without associated drugs to infer their ability to confer drug resistance, their relative fitness status in different environments, and their transmissibility across individuals.

Additionally, identifying transmission clusters is critical to recognizing groups who may be at risk of contracting HIV-1 or who may already be infected but are not yet aware of their diagnosis. Phylogenetic studies suggest that transmission clusters greatly contribute to the spread of HIV-1 within the population^47^; therefore, identifying high-risk groups, whether that is based on risk behavior or geographical location^48^, can help public health officials to better target prevention efforts and treatment options. The spread of infection is often associated with early HIV-1 infection^47^, consequently, molecular surveillance of the DC epidemic should continue in order to identify potential areas or clusters of transmission and, thus, help lower the HIV-1 incidence.

Towards this goal, we combined NGS sequence data with clinical characteristics to obtain a dynamic picture of the evolution of HIV-1 in the DC metro area. We detected high levels of clustering using haplotypes (32.8–64.2%, not including the *V1V2* region), as also seen in other HIV-1 cohorts (24–65%)^29,35,49–61^. Other studies, including one completed with Sanger sequencing in DC^28,31,36,52,62^, however, have found lower levels of clustering (7–17%), in agreement with those reported here for the consensus sequence phylogenetic clusters (*pol(c)* and *env(c)*) and the *V1V2* region for haplotypes (9.0%). Therefore, a more comprehensive understanding of HIV-1 transmission events in DC has been achieved when evaluating multiple genes together, rather than primarily focusing on polymerase genes that are typically screened for DRMs in clinical settings or used in investigations at the local health department level. By excluding envelope genes, informative transmission events can be missed, which could hinder community health prevention and intervention efforts. In an ideal setting, using all the genetic information available would be most favorable when investigating local HIV-1 phylodynamics.

In agreement with a recent study of HIV-1 transmission clusters in Chicago^59^, we also found association of risk factors within clusters. More HRH participants fell in our haplotype transmission networks compared to MSM, IDU, and participants with unknown risk (HRH = 23, MSM = 18, UNK = 11 each & IDU = 6). A total of 58.3% of the clusters that included an HRH participant also had an MSM participant. Likewise, a US study that included 12 major US cities^63^ found transmission clusters that contained overlap between participants who were MSM and HRH. Mixing of risk types in HIV-1 subtype B transmission clusters has also been observed in Switzerland, Iceland, and Nordic European countries^60,64,65^. Contrarily, Kouyos *et al*.^65^ found segregation based on location among individuals who were included in a transmission cluster despite having overlapping risk factors. Risk groups may be mixing due to underreporting of risk behaviors or bisexual behavior^65–67^. This heterogeneity of risk groups in transmission clusters suggests that focusing on individuals within city areas (e.g., wards in Washington, DC) to concentrate resources and information may help in addressing the HIV-1 epidemic.

Otherwise, we were unable to determine the mode of transmission for the “unknown modes of transmission” group (16.2% of our sample). Nonetheless, our results suggest that the mode of transmission may not be as important for prevention and intervention efforts as the location where transmission events are occurring. Likewise, Morgan *et al*.^59^ suggested not targeting efforts towards risk groups, but rather age groups, particularly younger people, in Chicago. The average age of the DC participants included in a haplotype transmission cluster was 46.6 years of age. If DC’s younger population is being the most affected, as suggested by the new cases identified by the DC DOH in 2016 and 2017^1,3^, taking a spatial dynamic approach to intervention with continued surveillance may help. Through surveillance studies, further adapted location-based prevention efforts can be employed.

Notably, our analysis has some limitations. We included only 68 participants of the approximately 10,000 people enrolled in the DC Cohort^3^, whereas past studies conducted in DC included 700 (Kassaye *et al*.) and 1,500 participants (Pérez-Losada *et al*.). However, the demographics in our sample size are similar to PLWH in DC^3,4^. As a prospective study conducted as part of an ongoing HIV-1 surveillance program associated with the DC Cohort, we capitalized on all the current cases that met our inclusion criteria (see Matierals and Methods: DC cohort). These past studies of HIV-1 diversity in Washington, DC were historical in nature and, therefore, had larger sample sizes available. Our study also applied a powerful next-generation sequencing approach instead of Sanger sequencing (previous DC studies), to characterize the current HIV-1 epidemic. With the implementation of NGS, mapping very diverse short reads to a reference genome poses alignment issues^14^, which can add difficulty to downstream analyses. We circumnavigated this alignment issue by using HAPHPIPE, where the reads were mapped against a tailored reference genome, thus resulting in higher alignment rates and fewer errors. Nonetheless, aligning very diverse reads still remains an issue. Although new sites under selection were identified using NGS, their clinical relevance as potential DRMs requires further validation. We also recognize that we used conservative genetic distance cutoff values for determining transmission clusters, which could result in lower numbers of transmission clusters^68^; however, this conservative estimate reduced the number of false positive transmission clusters. Finally, due to the nature of predicting transmission clusters and the potential for missing individuals, we are not able to determine the direction of infection within the transmission clusters. We were also unable to rule out batch effects or laboratory artifacts accounting for any transmission cluster which participants were included in the same sequencing run.

## Conclusions

This study showed that NGS and epidemiological data can be used to characterize the current phylodynamics of a subset of people living with HIV, enhancing our understanding of the diversity and local dynamics of the HIV-1 epidemic in the DC area. HIV-1 diversity in DC is high and seems to remain stable over time. Furthermore, NGS of the envelope gene provided sufficient coverage to compare transmission cluster inference across HIV-1 gene regions^14,20^. Additional transmission clusters were identified when using HIV-1 intra-host haplotypes instead of consensus sequences, which led to networks linking a higher number of participants. Moreover, transmission clusters varied across genes, with each gene suggesting a different transmission story. Hence, using multiple HIV-1 genes or whole genomes is recommended to infer more reliable transmission clusters. Inferred clusters should then be linked to locations in DC to target transmission intervention efforts. Additionally, HIV-1 drug resistance was only found when using haplotypes in a single young adult in our cross-sectional sample of the cohort. Future studies should also focus on age groups and geographic regions rather than only risk factors. As the DC area maintains significant rates of HIV-1 infection, integrating present and past molecular data from previous studies conducted in DC in 2017^29^ and 2013^28^ will help to paint a comprehensive picture of the HIV-1 transmission and evolution of drug resistance in this high prevalence urban U.S. city. Future HIV-1 phylodynamic studies should also include more participants, particularly young adults, and newly diagnosed persons to provide a comprehensive view of DRM prevalence in treatment-naïve individuals in the DC area. Studies revealing the severity of transmitted drug resistance in the DC population may provide physicians and public health workers with additional information to design more effective treatment plans for newly diagnosed individuals and intervention strategies for targeted key populations.

## Materials and Methods

### Ethics

Institutional Review Board (IRB071029) approval was obtained from The George Washington University IRB (which serves as the IRB of Record for eight of the participating sites), the DC DOH IRB, and the remaining site IRBs. Informed consent was obtained and documented prior to conducting study procedures. Sample collections from participants were performed in accordance with relevant guidelines and regulations.

### DC cohort

Participants from the DC Cohort were recruited for this molecular epidemiology sub-study from January 2016 through May 2017. Eligibility criteria included current DC Cohort enrollment, ≥18 years of age, HIV-1 diagnosis within prior 12 months of enrollment or detectable HIV-1 viral load of ≥1,500 copies/mL, ability to provide written informed consent, and completion of a behavioral survey; a total of 104 participants met the eligibility criteria. Blood samples were collected at the clinical sites and transported to George Washington University for processing, targeted amplification, library preparation and NGS. Sample sequences were paired with clinical and demographic data retrieved from the database from the DC Cohort (Table 1). Clinical and demographic characteristics collected included age, race/ethnicity, sex at birth, gender, country of birth, state of residence, zip code, HIV-1 risk factor, presence of co-infections (e.g., chlamydia, gonorrhea, syphilis, trichomoniasis, and Hepatitis B and C), duration of infection, CD4 count, viral load, ART exposure, ART regimen type, date of sample, and date of HIV-1 diagnosis. The paired data were de-identified and analyzed using the approaches described below.

### Next-Generation sequencing

Total RNA was extracted from each patient’s plasma sample, and cDNA synthesis followed. The QIAamp Viral RNA Mini Kit (Cat. #52904, Qiagen, Gaithersburg, MD) and the SuperScript^™^ IV First-Strand Synthesis System (Cat. # 18091050, Invitrogen, Carlsbad, CA) were used respectively and according to manufacturers’ instructions. Multiple sets of HIV-1 specific primer pairs were used to target and amplify using polymerase-chain-reaction (PCR) the protease (*PR*), reverse transcriptase (*RT*), integrase (*int*), and envelope (*env*) HIV-1 genes (~43% of genome)^36^. Library preparation was completed with Nextera XT Library Prep (Cat. # 15032350, Illumina, Dan Diego, CA). Samples were then sequenced on eight runs on an Illumina MiSeq platform using the MiSeq v2 (300 cycles) chemistry (Cat. # MS-102–2002, Illumina). Both library prep and sequencing were completed according to the manufacturer’s instructions. All DNA sequence files are available from the GenBank database under SRA accession: PRJNA517147.

### Sequence analyses

The raw sequence data for each patient were processed through HAPHPIPE (https://github.com/gwcbi/haphpipe), a HAplotype reconstruction and PHylodynamics PIPEline for genome-wide assembly of viral consensus sequences and haplotypes^69^. Briefly, HAPHPIPE includes modules for quality trimming, error correction, assembly, and haplotype reconstruction. We put the raw sequencing FASTQ files through quality control and quality trimming with Trimmomatic^70^. Error correction of the reads was completed with an earlier version of HAPHPIPE that used BLESS^71^, and the cleaned reads were mapped against the current HIV-1 subtype B reference sequence HXB2 (Genbank accession: K03455)^72^. Through iterative refinement, the cleaned reads were then mapped back to the reference sequence generated in the mapping step with Bowtie2^73^. This iterative refinement step was completed twice, first using only a random subsampling of the reads (25% subsampling) and the fast-local mapping option to speed up the computational time, and second using all of the sequence reads and the very sensitive mapping option to further refine the individually crafted reference sequence. A consensus sequence was generated from the refined reference sequence, and a final refinement step was concluded with BLAST^74^ against this refined consensus sequence. The resulting sequences were filtered to include participants that contained a passing amplicon, defined as having > 95% of the amplicon covered by 10x or greater read coverage. Amplicons that did not pass this filter were removed, and this subset was then used for subsequent phylodynamic analyses (Table 1).

Sequence data for each PCR amplicon (*PR/RT, int*, and *env)* were aligned individually using MAFFT with the L-INS-i algorithm^75^ in Geneious (ver. 9.1.6)^76^. Protease (*PR*) and reverse transcriptase (*RT*) were extracted from the *PR/RT* amplicon, and *env* was further divided into the variable regions: gp120 *V1V2* (HXB2 coordinates: 6615–6812) and gp120 *V3* (HXB2: 6984–7349). *PR*, *RT*, and *int* were concatenated into the *pol(c)* gene region, and *V1V2* and *V3* were concatenated into the *env(c)* gene region. Concatenated gene regions will be distinguished from whole gene regions by adding “*(c)”* to the end of the gene name. Each gene (*PR*, *RT*, *int*, V1V2, and *V3*) was extracted from the amplicon data to fulfill different purposes: (1) remove any nucleotides belonging to other genes, for example the *env* PCR amplicon contained primer sequences, and therefore nucleotides belonging to the *vpu* gene region; (2) simulate amplicon sizes that could be covered end to end by paired-end reads to be consistent and comparable to future NGS studies with HIV-1 when using PrimerID or other local haplotype phasing techniques^8,77^; and (3) account for differences in PCR performance between and within samples by extracting a common, high-coverage region. Therefore, missing data in this dataset were low, and often only due to amplification failure of an entire amplicon. Concatenating the genes into their respective gene regions (*pol* and *env*) retained variants in genes that are often studied, such as protease and reverse transcriptase for drug resistant mutations. It also allowed comparisons to past studies based on Sanger sequencing that used either parts of genes or whole genes. Our overall goal was to keep the integrity of the individual genes while using as much of the NGS data as possible.

### Identification of subtypes and drug resistant mutations

HIV-1 subtype identification was completed for each concatenated gene region (*pol(c)* and *env(c)*) using the REGA subtyping tool (version 3)^78,79^. A total of 170 subtype reference sequences from the Los Alamos HIV-1 database (LANL; http://www.hiv.lanl.gov/) were included to assign the patient sequences to a particular subtype clade and validate the findings from REGA using phylogenetic methods described below. Drug resistant mutations were identified aligning the consensus concatenated gene nucleotide sequences with reference strains in the Stanford HIV Drug Resistance Database (https://hivdb.stanford.edu) using the HIVdb program^80^. Nucleotide positions under positive selection were identified using Fast Unconstrained Bayesian AppRoximation (FUBAR)^81^ in HyPhy^82^. Recombination in our HIV-1 data was accounted for with GARD^83,84^.

### Phylogenetic analyses

Phylogenetic estimations were completed for each concatenated gene region. The best-fit model of molecular evolution^85^ was estimated for each *pol(c)* and *env(c)* from the data using jModelTest2^86^ in CIPRES Science Gateway^87^. Amino acid positions corresponding to identified DRMs described above were removed prior to phylogenetic estimations to avoid potential bias due to selection. A maximum likelihood phylogenetic estimate using RAxML^88^ was made for each region with the 3 codon-position partitions^89^. The branch support for the RAxML phylogenetic trees was estimated with a bootstrap approach with 1,000 replicates^90^. Bayesian trees were inferred using MrBayes^91^. Four Markov chains (one cold and three heated) were run for 8 × 10^8^ generations sampling every 2,000 steps for each gene region, and each run was repeated twice. The output was analyzed in Tracer^92^ to assess convergence and mixing of the chains. Subtypes references for subtype D (GenBank accessions: K03454, AY371157, AY253311, U88824) and circulating recombinant forms CRF28,42-BF (GenBank accessions: FJ213781, FJ358521, FJ670529) and CRF10-CD (GenBank accessions: AF289548, AF289549, AF289550) were pulled from LANL and used as proper outgroups for the phylogenetic analyses^93^. Additional *RT* sequences from DC^29^ were included to observe how our data related to other DC sequences. We visualized the epidemiological data on the resulting trees with the Interactive Tree of Life (iTOL).

### Haplotype reconstruction

For the identification of transmission clusters and testing for associations of clinical variables to transmission clusters, it is ideal to characterize within patient viral variation as individual sequence variants (haplotypes) instead of combining all of the individual reads into a single consensus sequence^94,95^. Therefore, haplotypes for each patient were predicted from the sequence data using HAPHPIPE’s haplotype stages. Haplotype reconstruction was performed on each *PR/RT*, *int*, and *env* targeted PCR amplicons using PredictHaplo^96^. Each gene region (*PR*, *RT*, *V1V2*, and *V3*) was then extracted from the corresponding targeted amplicon and, using the methods described below, transmission clusters were estimated using the predicted haplotypes. No concatenation of the individual genes to form the regions *pol(c)* and *env(c)* was done with the haplotypes.

### Identification of transmission clusters

Transmission clusters were assessed for each *pol(c)* and *env(c)*, as well as for each of the gene regions with the haplotypes, using phylogenetic methods^89,91^ and the genetic-distance based clustering method HIV-TRACE^97^. Phylogenetic transmission networks were defined as clades with bootstrap proportions ≥70 or posterior probabilities ≥95%. Genetic distance thresholds of 0.01^29,62^ and 0.02^98^ substitutions/site were used for *pol* and *env* in HIV-TRACE, respectively, to identify potential transmission events. Ambiguities were handled with the HIV-TRACE option “average” to avoid biases and false positives and minimum overlap was 1/genetic distance threshold and adjusted for size of amplicon, as recommended. Default settings were used for the remaining parameters. Transmission clusters were compared between gene regions.

### Diversity estimation

Haplotype diversity (h), the number of segregating sites (S), nucleotide diversity (π), and Watterson’s genetic diversity (θ) (see^99^) were estimated for both the consensus *pol(c)* and *env(c)* regions per patient using DnaSP (ver. 6.11.01)^100^. Haplotype diversity (h), which takes into account the number of haplotypes and their relative frequencies, was also estimated from PredictHaplo results according to Nei and Tajima^101^. Both diversity estimates were used as the number of haplotypes estimated from DnaSP is representative of inter-patient diversity, whereas the number of haplotypes and haplotype diversity estimates from the PredictHaplo results are representative of intra-patient diversity. Significance for haplotype diversity between clinical variables was measured with the Wilcoxon rank sum test or Kruskal-Wallis test in R v 3.6.0^102^ using RStudio v 1.2.1335^103^.

### Ethical approval

Written informed consent was obtained from all participants prior to enrollment in the DC Cohort and the molecular epidemiology sub-study. The DC Cohort and molecular epidemiology studies were approved by the Institutional Review Board at The George Washington University, which serves as the IRB of record for Whitman-Walker Health, La Clinica del Pueblo, Family and Medical Counseling Service, Unity Health Care, The GW Medical Faculty Associates, MetroHealth, and Children’s National Health System (pediatric and adolescent clinics). The study was independently approved by the IRBs of record at Howard University Hospital (adult and pediatric clinics), MedStar Washington Hospital Center, Georgetown University, and the Veterans Affairs Medical Center.

## Supplementary information

Supplemental Figure 1.
