# Author Correction: A cross-sectional study to characterize local HIV-1 dynamics in Washington, DC using next-generation sequencing

**DOI:** 10.1038/s41598-020-63859-y

**Published:** 2020-04-22

**Authors:** Keylie M. Gibson, Kamwing Jair, Amanda D. Castel, Matthew L. Bendall, Brittany Wilbourn, Jeanne A. Jordan, Keith A. Crandall, Marcos Pérez-Losada, the DC Cohort Executive Committee, Thilakavathy Subramanian, Jeffery Binkley, Rob Taylor, Nabil Rayeed, Cheryl Akridge, Stacey Purinton, Jeff Naughton, Natella Rakhmanina, Larry D’Angelo, Michael Kharfen, Angela Wood, Michael Serlin, Princy Kumar, David Parenti, Alan Greenberg, Anne Monroe, Lindsey Powers Happ, Maria Jaurretche, James Peterson, Ronald D Wilcox, Sohail Rana, Michael A Horberg, Ricardo Fernández, Annick Hebou, Carl Dieffenbach, Henry Masur, Jose Bordon, Gebeyehu Teferi, Debra Benator, Maria Elena Ruiz, Deborah Goldstein, David Hardy

**Affiliations:** 1 Computational Biology Institute, The Milken Institute School of Public Health, The George Washington University, Washington, DC 20052 USA; 2 Department of Epidemiology, The Milken Institute School of Public Health, The George Washington University, Washington, DC 20052 USA; 3 Department of Biostatistics and Bioinformatics, The Milken Institute School of Public Health, The George Washington University, Washington, DC 20052 USA; 4 CIBIO-InBIO, Centro de Investigação em Biodiversidade e Recursos Genéticos, Universidade do Porto, Campus Agrário de Vairão, Vairão, Portugal; 5 Cerner Corporation, Arlington, VA 22209 USA; 6 Children’s National Medical Center Pediatric Clinic, Washington, DC 20010 USA; 7 Children’s National Medical Center Adolescent Clinic, Washington, DC 20010 USA; 8 DC Department of Health HAHSTA, Washington, DC 20002 USA; 9 Family and Medical Counseling Service, Washington, DC 20020 USA; 10 Georgetown University, Washington, DC 20007 USA; 11 George Washington Medical Faculty Associates, Washington, DC 20037 USA; 12 Howard University Hospital Adult Clinic, Washington, DC 20059 USA; 13 Howard University Hospital Pediatric Clinic, Washington, DC 20060 USA; 14 Kaiser Permanente, Rockville, MD 20852 USA; 15 La Clinica Del Pueblo, Washington, DC 20009 USA; 16 MetroHealth, Washington, DC 20005 USA; 17 National Institutes of Health, Bethesda, Maryland 20892 USA; 18 Washington Health Institute, Washington, DC 20017 USA; 19 Unity Health Care, Washington, DC 20019 USA; 20 Veterans Affairs Medical Center, Washington, DC 20422 USA; 21 Washington Hospital Center, Washington, DC 20010 USA; 22 Whitman-Walker Health, Washington, DC 20036 USA

Correction to: *Scientific Reports* 10.1038/s41598-020-58410-y, published online 06 February 2020

This Article contains errors in Table 3. In this Article, data values provided for Participant 16 are incorrect. The correct Table 3 appears below as Table 1.

**Table 1:** Haplotype diversity estimates from PredictHaplo results.

Participant	*PR/RT*		*int*		*env*		Viral Load (copies/mL)	ARV Exposure	ARV Regimen Type
	Number of Haplotypes	Haplotype Diversity	Number of Haplotypes	Haplotype Diversity	Number of Haplotypes	Haplotype Diversity			
8	NA	NA	1	0	NA	NA		E	2 NRTI+1 ENH+1 INSTI
9	NA	NA	5	0.066	11	0.038	476	E	
12	1	0	4	0.727	2	0.393	77	E	2 NRTI+1 NNRTI
13	NA	NA	1	0	2	0.302		N	
16	3	0.33	1	0	3	0.415		E	
18	NA	NA	1	0	NA	NA	119149	E	
19	2	0.436	2	0.499	4	0.721	244	N	
20	2	0.479	1	0	2	0.281	5663	E	1 NRTI+1 PI+1 ENH
23	3	0.352	2	0.484	NA	NA	927	E	2 NRTI+1 ENH+1 INSTI
25	1	0	1	0	NA	NA		E	2 NRTI+1 ENH+1 INSTI
26	2	0.452	2	0.466	NA	NA		N	
27	1	0	1	0	NA	NA	11	E	1 PI+1 ENH
29	2	0.464	2	0.25	5	0.62	1	E	2 NRTI+1 ENH+1 INSTI
30	2	0.441	3	0.579	1	0	625	E	
31	3	0.64	3	0.526	2	0.312		E	2 NRTI+1 INSTI
32	2	0.146	2	0.339	2	0.498		E	2 NRTI+1 NNRTI+1 PI+1 ENH
33	1	0	3	0.563	3	0.601		E	2 NRTI+1 ENH+1 INSTI
34	2	0.429	2	0.2	1	0		E	2 NRTI+1 INSTI
35	2	0.385	1	0	3	0.446		E	2 NRTI+1 INSTI
37	2	0.274	1	0	10	0.027	81	E	
39	NA	NA	1	0	NA	NA		E	2 NRTI+1 ENH+1 INSTI
40	2	0.494	4	0.692	3	0.623		E	
42	1	0	2	0.215	2	0.445	13979	E	2 NRTI+1 ENH+1 INSTI
43	1	0	7	0.833	NA	NA	343	E	
45	1	0	10	0.704	NA	NA		E	2 NRTI+1 ENH+1 INSTI
46	2	0.153	3	0.306	2	0.5		N	
47	2	0.263	2	0.42	1	0	2755	E	1 NNRTI+1 PI+1 ENH+1 INSTI
49	2	0.455	1	0	2	0.324	282	E	2 NRTI+1 ENH+1 INSTI
50	1	0	2	0.344	3	0.624	576	E	2 NRTI+1 PI+1 ENH
51	1	0	1	0	3	0.593		E	2 NRTI+1 PI+1 ENH+1 INSTI
52	2	0.439	1	0	NA	NA		E	2 NRTI+1 PI+1 ENH
54	3	0.583	1	0	2	0.491	412	E	2 NRTI+1 PI+1 ENH
55	1	0	1	0	2	0.384		E	1 PI+1 ENH
56	12	0.392	3	0.641	NA	NA	1368	E	2 NRTI+1 ENH+1 INSTI
57	1	0	1	0	NA	NA	12786	E	2 NRTI+1 ENH+1 INSTI
58	1	0	3	0.59	4	0.659	1281	E	2 NRTI+1 ENH+1 INSTI
59	3	0.572	1	0	4	0.705	581	E	2 NRTI+1 INSTI
60	2	0.487	3	0.619	4	0.716		E	2 NRTI+1 PI+1 ENH
61	2	0.27	3	0.632	4	0.679	432	E	
63	3	0.612	6	0.708	1	0	57	E	2 NRTI+1 PI
64	5	0.759	3	0.576	4	0.697		E	
65	2	0.492	2	0.441	3	0.186		E	2 NRTI+1 PI+1 ENH
66	1	0	2	0.49	5	0.602	32	E	2 NRTI+1 ENH+1 INSTI
67	NA	NA	2	0.45	5	0.767		E	2 NRTI+1 NNRTI
68	3	0.564	5	0.736	5	0.758		E	2 NRTI+1 NNRTI
69	1	0	4	0.543	7	0.777	48	E	2 NRTI+1 PI+1 ENH
70	4	0.603	2	0.364	5	0.743	17	N	
71	1	0	4	0.72	6	0.812		E	1 NRTI+1 NNRTI+1 INSTI
72	2	0.209	3	0.614	NA	NA	353	E	2 NRTI+1 NNRTI
73	1	0	3	0.66	2	0.414		E	2 NRTI+1 ENH+1 INSTI
74	1	0	5	0.779	4	0.037	1	E	2 NRTI+1 ENH+1 INSTI
75	1	0	3	0.533	7	0.663		E	2 NRTI+1 PI+1 ENH
76	1	0	1	0	5	0.777	432	E	2 NRTI+1 INSTI
77	4	0.613	4	0.658	6	0.768	113	E	2 NRTI+1 PI+1 ENH+1 INSTI
78	2	0.183	3	0.608	5	0.664	113	E	2 NRTI+1 PI+1 ENH
79	1	0	1	0	2	0.396		E	2 NRTI+1 INSTI
82	1	0	NA	NA	NA	NA		N	
83	2	0.467	NA	NA	NA	NA	554	E	2 NRTI+1 NNRTI
85	2	0.351	1	0	2	0.477	183	E	2 NRTI+1 INSTI
86	2	0.433	1	0	NA	NA		E	2 NRTI+1 PI+1 ENH+1 INSTI
87	2	0.499	NA	NA	NA	NA		E	2 NRTI+1 ENH+1 INSTI
88	1	0	NA	NA	1	0	37	N	
90	3	0.534	2	0.498	3	0.54	8914	E	2 NRTI+1 PI+1 ENH+1 CCR5+1 INSTI
91	1	0	2	0.003	NA	NA	117	E	2 NRTI+1 INSTI
93	2	0.446	1	0	NA	NA	94	E	2 NRTI+1 ENH+1 INSTI
94	1	0	NA	NA	NA	NA	145	E	2 NRTI+1 PI+1 ENH
97	5	0.718	2	0.455	5	0.782		E	2 NRTI+1 INSTI
99	1	0	NA	NA	1	0	184	E	2 NRTI+1 ENH+1 INSTI
Avg	2	0.265	2	0.357	4	0.47			

A haplotype diversity of 0 indicates no diversity because only a single haplotype was reconstructed by PredictHaplo for the sample. Amplicons that did not pass the filtering thresholds for a sample are indicated by “NA”. ARV exposure is reported at time that blood sample was taken. N: Naïve, E: Experienced, NRTI: Nucleoside reverse transcriptase inhibitors, NNRTI: Non-nucleoside reverse transcriptase inhibitors, ENH: enhancer elements, PI: Protease Inhibitor, INSTI: Integrase Strand Transfer Inhibitor, CCR5: Cysteine-Cysteine Chemokine Receptor 5.

