# Supplementary material for: A cross-sectional study to characterize local HIV-1 dynamics in Washington, DC using next-generation sequencing
Source: Sci Rep. 2020 Feb 6;10:1989. doi: 10.1038/s41598-020-58410-y (PMC7004982; doi:10.1038/s41598-020-58410-y)

A cross-sectional study to characterize local HIV-1 dynamics in Washington, DC using next-generation sequencing

Keylie M. Gibson[1*], Kamwing Jair[2], Amanda D. Castel[2], Matthew L. Bendall[1], Brittany Wilbourn[2], Jeanne A. Jordan[2], Keith A. Crandall[1,3], Marcos Pérez-Losada[1,3,4] and the DC Cohort Executive Committee[†]

[1]Computational Biology Institute, The Milken Institute School of Public Health, The George Washington University, Washington DC 20052, USA. [2]Department of Epidemiology, The Milken Institute School of Public Health, The George Washington University, Washington DC 20052, USA. [3]Department of Biostatistics and Bioinformatics, The Milken Institute School of Public Health, The George Washington University, Washington DC 20052, USA. [4]CIBIO-InBIO, Centro de Investigação em Biodiversidade e Recursos Genéticos, Universidade do Porto, Campus Agrário de Vairão, Vairão, Portugal

* Corresponding author
Email: kmgibson@gwu.edu (KMG)
[†]Membership of the DC Cohort Executive Committee is provided in the Acknowledgments.

Supplemental Figure 1. Phylogeny of DC HIV-1 *pol* sequences. Participants from our study are colored in red and are seen distributed throughout the tree and among past DC HIV-1 sequences.

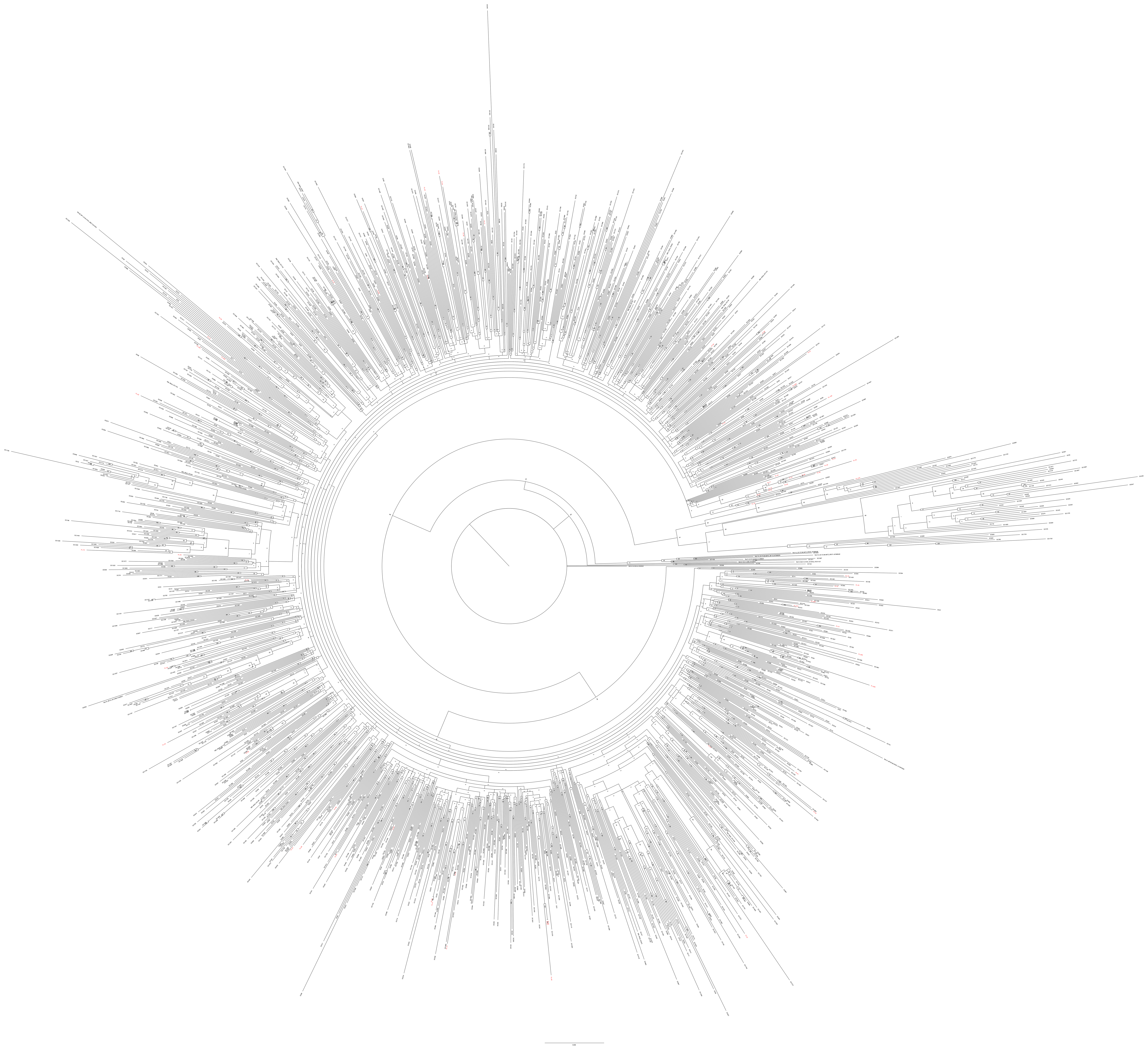

Supplement: Supplementary file 1 — Supplemental Figure 1. [file 41598_2020_58410_MOESM1_ESM.pdf]
